# Corrosion Behaviour of High-Pressure Die-Cast AZ91 Alloy in NaCl Solution: Effects of Friction Stir Process at High Rotational Speed

**DOI:** 10.3390/ma16206620

**Published:** 2023-10-10

**Authors:** Emanuele Ghio, Emanuela Cerri

**Affiliations:** Department of Engineering and Architecture, University of Parma, Via G. Usberti, 181/A, 43124 Parma, Italy; emanuela.cerri@unipr.it

**Keywords:** AZ91 alloy, pitting corrosion, friction stir process, corrosion resistance, surface roughness, micro-galvanic corrosion, high-pressure die cast

## Abstract

The AZ series of Mg alloys have become promising in several industrial fields thanks to its potential microstructure refinement and the β-Mg_17_Al_12_ eutectic that controls the mechanical behaviour. Simultaneously, the rapid degradation characterizing Mg alloys makes the investigation of their corrosion behaviour necessary. The present work considers high-pressure die cast (HPDC) AZ91 alloy to evaluate its corrosion behaviour in 1M NaCl solution and investigates how different friction stir process parameters can affect the corrosion responses. No studies analyse the effects induced by the friction stir processed zone, reached using high rotational speeds (>2000 rpm), on the unprocessed HPDC AZ91 alloy. In addition, the morphological analysis of the corroded surfaces having a friction stir processed zone, in which the grain refinement was not obtained, is not present in the literature yet. Microstructural features were investigated by optical microscope and X-ray diffraction analysis before and after the friction stir process. These were subsequently correlated to the corrosion responses after the immersion tests. The results show that HPDC samples with a very smooth surface have the best corrosion resistance with a corrosion rate lower than 3 mm/year, evaluated through the weight loss, compared to the rougher ones. Both the amount of β-Mg_17_Al_12_ eutectic and the wt.% Al in the α-Mg matrix, as well as the surface roughness, influence the corrosion behaviour of friction stir processed samples. The best corrosion resistance was obtained with an HPDC alloy processed at 2500 rpm and 50 mm/min.

## 1. Introduction

Magnesium alloys are demonstrated to be attractive to the automotive sector, in which the AZ91 alloy covers 90% of all Mg cast products, and aerospace applications thanks to their high specific strength and low density which promote, for example, a decrease in oil consumption [1]. Simultaneously, their excellent biocompatibility and good biomechanical compatibility make them suitable in the biomedical field, e.g., for orthopaedic implants or the treatment of cardiovascular disease [2,3,4,5]. Magnesium-based alloys are, however, susceptible to corrosion attacks in humid or wet atmospheres after an oxide layer rupture. Presently, the following oxidation–reduction reaction has been accepted by the literature overview to describe the corrosion behaviour of Mg alloys [2,6]:

Mg + 2H_2_O → Mg(OH)_2_ + H_2_

where the anodic and the cathodic reactions are represented by the following half reactions, respectively:

Mg → Mg^2+^ + 2e^−^

H_2_O + 2e^−^ → H_2_ + 2OH^−^

Corrosion reactions are amplified in the marine environment where the concentration of Cl^−^ damages the passive layer, triggers pitting corrosion, and consequently increases the corrosion current regardless of pH level [7]. In fact, Cl^−^ ions can easily penetrate within the oxide layer and can promote cation diffusion due to their significant solubility and small radius. These phenomena increase from 3.5 wt.% NaCl (seawater-like solution) to 7 wt.% in the corrosive environment [8,9]. Concerning the chemical composition of the AZ series, alloying elements like Al and Zn were added, firstly, to improve the mechanical properties and, secondly, to modify the corrosion behaviour of the AZ series. Warner et al. [10] showed beneficial effects on corrosion resistance already with an Al content of 5%, whereas Froes et al. [11] observed an improvement in corrosion resistance with a content of 9%. At the same time, low degradation rates are ensured by a wt.% of Zn lower than 3% [2]. As regards AZ91 alloy, the 9wt.% Al and the 1wt.% Zn promote higher corrosion resistance than pure Mg or alloys like AZ31 and AZ81 [2,12,13]. On the other hand, the interdendritic network, exhibited by the AZ91 alloy and formed by the β-Mg_17_Al_12_ eutectic in the α-Mg matrix, greatly influences the corrosion behaviour [6,14]. The micro-galvanic couple between the eutectic (E^0^ = −1.25 eV) and the α-Mg matrix (E^0^ = −1.6 eV) accelerates the corrosion phenomena. On the other hand, the morphology and distribution of the β phase can modulate the corrosion propagation because it can act as a corrosion barrier [6,15,16]. In a microstructure where the β phase forms a network structure, corrosion is slowed down thanks to its barrier effect, whereas the corrosion is accelerated by coarse and isolated β phase in the α-Mg matrix [17,18]. Lastly, grain size affects the corrosion properties. Some studies [4,16,19,20] affirmed that a grain size refinement improved the corrosion resistance of the Mg alloys in neutral and alkaline NaCl solutions due to the ability of oxide layer formation, while other ones [21,22] underline a deterioration.

The corrosion behaviour of the AZ91 alloy is therefore influenced by the severe and localized plastic deformation induced by the friction stir processing (FSP) due to both the grain refinement, which increases strength, ductility, and deformation at high temperatures, and the variation of the β-Mg_17_Al_12_ morphology [15,23,24]. Given the importance of the degradation rate of Mg alloys, the present manuscript aims to evaluate the corrosion performance of HPDC AZ91 samples that were friction stir processed (FSPed) at high rotation rates. In the current literature overview [25,26,27,28,29], few studies are based on these aspects. Saikrishna et al. [27] analysed the corrosion behaviour of FSPed AZ91 samples after annealing treatment at 340 °C and revealed an increment of the corrosion rate in a 0.9% NaCl solution. Sidhu et al. [28] evaluated the corrosion performance of AZ91 samples FSPed with two different-shaped non-consumable tools. No significant results were obtained in terms of corrosion rate (mm/year) by immersing the sample in 3.5wt.% NaCl solution for 72 h. Hasani et al. [29] affirmed that FSP can decrease the corrosion resistance of AZ91 cast samples, which were FSPed with several pass numbers, in a 3.5% NaCl solution. Also considering other studies [17,29,30,31,32], in which the rotational speed is always lower than 2000 rpm, no one is focused on the AZ91 alloy produced by HPDC and FSPed at a high rotation rate (>2000 rpm) as in the present manuscript. Finally, it is useful to underline that the modern HPDC technology methods are very good manufacturing processes in terms of their high capacity and efficiency. They also allow for manufacturing parts characterized by a high surface quality, fine microstructure thanks to the fast cooling rate, and thin-walled and complex geometry [33,34].

The current study analyses the corrosion behaviour of HPDC AZ91 samples both in unprocessed conditions and after the FSP that was performed at different high rotation rates (2500, 3000 rpm) and advancing speeds (30, 50 mm/min). The paper first evaluates the corrosion rates (mm/year) occurring in a NaCl solution at room temperature and, secondly, analyses how the several effects induced on the microstructure by the FSP parameters influence the corrosion behaviour of HPDC AZ91 alloy. Lastly, the influence of surface roughness on the corrosion behaviour of unprocessed and FSPed AZ91 samples is also studied.

## 2. Materials and Methods

### 2.1. Sample Preparation

**Figure 1:**
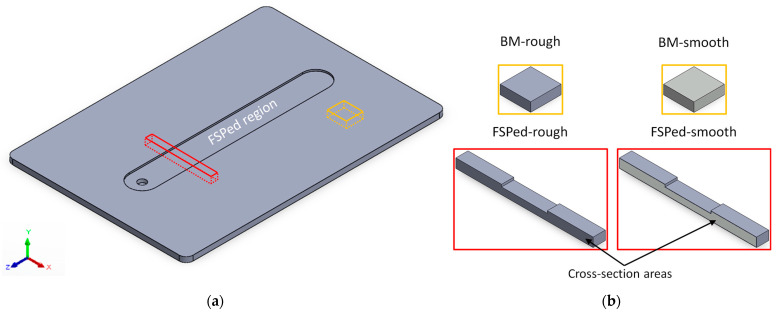
Schematic representation of AZ91 plate FSPed with the process parameters listed in Table 2 (**a**), where orange and red lines indicate the unprocessed platelets and FSPed samples (**b**), respectively, used for microstructural investigations and corrosion tests. The investigated corroded surfaces in FSPed samples are the cross-sectional areas indicated by the black arrows.

A high-pressure die-cast AZ91 magnesium alloy, whose chemical composition is listed in Table 1, was provided in plates with a thickness of 3 mm (Figure 1).

Plates were friction stir processed through an H13 tool steel at high rotation rates (ω_1_ = 2500 rpm and ω_2_ = 3000 rpm) and advancing speeds (*v*) of 30 and 50 mm/min. The truncated cone-shaped tool had a diameter of 15 mm and a height of 2.3 mm. A K-type thermocouple was used to measure the temperatures (Table 2) behind the advancing and rotating pin during the FSP.

Several samples were cut from the plates to investigate both the microstructural features and the corrosion behaviour in the unprocessed (platelets: orange lines, Figure 1a) and in FSPed (FSPed: red lines, Figure 1a) conditions. Platelets were only formed by the base material (BM) and characterized by dimensions of 10 × 10 × 3 mm^3^, while the FSPed ones (45 × 4 × 3 mm^3^) were formed by the FSPed region surrounded by BM. In both cases, samples were indeed considered in the as-cast (rough, grey areas in Figure 1b) condition, namely with the surface finishing impresses from the mould during the cast process, and after the grinding process (smooth, light grey areas in Figure 1b). Grinding operations were performed by using abrasive discs (SiC paper), from P80 to P4000, on all surfaces of the unprocessed samples and on the cross-section of the FSPed ones after the cut process.

### 2.2. Surface and Microstructural Characterizations

Surface roughness of both BM platelets and FSPed samples was measured through an optical 3D profilometer (Taylor Hobson, Leicester, UK) on an area of 3 × 3 mm^2^ in rough and smooth conditions, respectively (Figure 1b).

Surface quality of FSPed regions of the AZ91 plates was investigated through a stereo microscope (StereoBlue, Euromex, Arnhen, The Netherlands).

The microstructure on the cross-section area of FSPed samples (red lines, Figure 1b) was investigated by an optical microscope (DMi8 Leica, Wetzlar, Germany). The samples were mechanically ground and polished using a 1 μm diamond polishing suspension. They were then etched by the Picral etchant (5 mL acetic acid, 25 mL ethanol, 25 mL distilled water, and 2 g picric acid). X-ray diffraction (XRD) analysis was performed using a Cuα radiation tube working at 30 mA and 40 kV, while α-Mg grain size was measured by linear intercept method.

The volume fraction $\left(V_{f,\beta }\right)$ of β-Mg_17_Al_12_ eutectic was calculated using the following Equation (1):

(1) $$V_{f,\beta }=\frac{A_{\beta }}{\sum_{i}A_{i}}$$

where $A_{\beta }$ is the total integrated area of the β-Mg_17_Al_12_ eutectic and $\sum_{i}A_{i}$ is the total area of all detected phases. The integrated areas were evaluated by applying the Pearson VII function in Origin Pro 2023 [35]. In-depth analyses of the microstructure features and mechanical properties were discussed in our previous works [23,36,37].

### 2.3. Corrosion Behaviour: Immersion Test

The corrosion behaviour of unprocessed platelets and FSPed samples was analysed through immersion tests in 1M NaCl solution, which was kept constant at room temperature, for the exposure time in the range from 1 h to 160 h. The high concentration of Cl^−^ ions in the bath (lab-grade NaCl + distilled water) was used to simulate the highly corrosive environments in which the halide ions damage the passive film and cause pitting corrosion. In addition, 1M NaCl accelerates the corrosion behaviour maintaining an adequate oxygen concentration in the bath [17,36,37]. These aggressive conditions, and the strong localized corrosive attack induced in AZ91 samples, helped the analysis of the corrosion behaviour in FSPed regions. Simultaneously, they helped the corrosion behaviour analysis in FSPed regions (areas indicated by the black arrows in Figure 1b) due to the intensification of the corrosion phenomena. The corrosion rate (CR, (mm/year)) was evaluated, according to both the ASTM NACE TM0169/G31 standard [38] and other studies [3,13,27], by the following equation:

(2) $$CR=k\frac{∆W}{tA\delta }$$

where the constant k (-) is equal to 87.6 (W) to obtain a CR in mm/year, ∆W (mg) is the weight loss, t (h) is the immersion time, A (cm^2^) is the area of the sample, and δ is the density of magnesium alloy (1.81 g/cm^3^). The weights of unprocessed and FSPed samples were measured before and after each immersion test by an analytical balance. Each sample extracted from the NaCl solution was then immersed in a 3.3 M acetic acid solution for 10 s to remove the corrosion products [39], rinsed in distilled water, and dried before each weighing. Corrosion behaviour was also correlated to the surface finishing of the unprocessed and FSPed samples (Figure 1).

## 3. Results and Discussion

**Figure 2:**
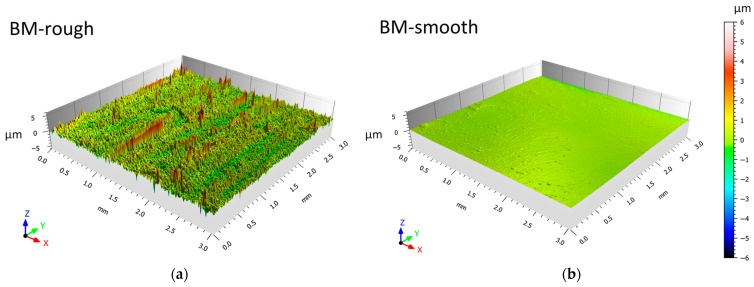
The 3D surface roughness maps of HPDC AZ91 before (**a**) and after (**b**) the grinding process.

Figure 2 shows the 3D surface maps of the unprocessed platelet (Figure 1b) samples before (Figure 2a) and after (Figure 2b) the grinding process that reduces their areal surface roughness from 0.45 ± 0.01 μm (BM-rough) to 0.07 ± 0.01 μm (BM-smooth). Consequently, S_z_ decreases from ~16 μm to ~2 μm. Also considering the cross-section areas of FSPed samples (Figure 1b), rough and smooth conditions are characterized by S_a_ values of 0.47 ± 0.01 μm and 0.08 ± 0.01 μm, respectively. Therefore, the cutting process, performed to obtain BM platelets and FSPed samples (Figure 1b), and the mould surface impressed comparable surface roughness values on AZ91 samples.

**Figure 3:**
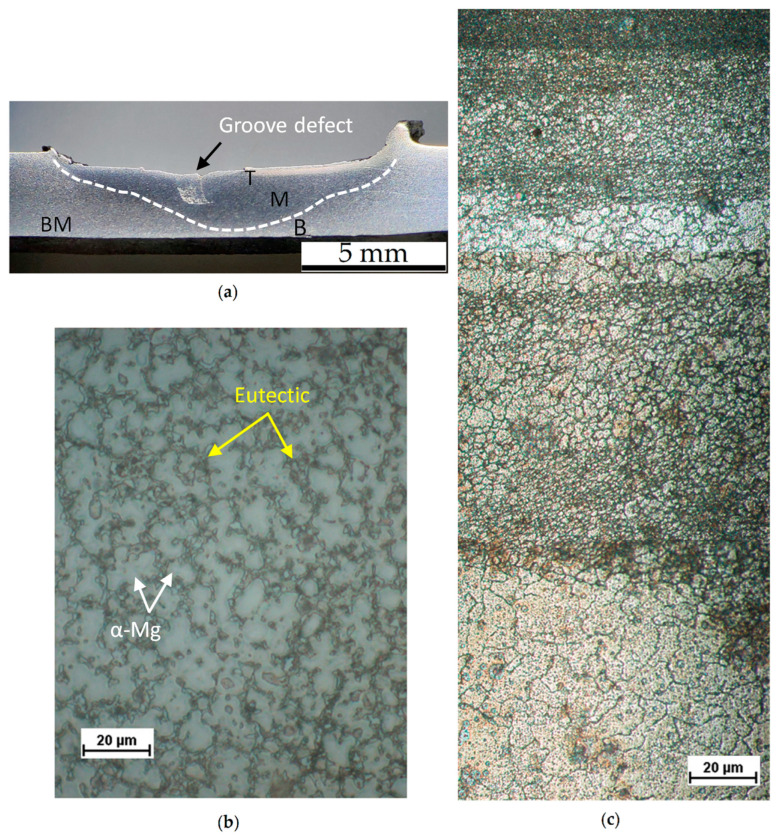
(**a**) Cross-section of 2.5-30 sample where T, M, and B indicate the top, middle, and bottom regions, respectively. White dotted line divides the FSPed region and the BM. (**b**,**c**) Optical microscope images of the BM and top region of the SZ.

Figure 3a is a macrograph showing the lack-of-fill defect zone (i.e., groove defect) and the FSPed region (dotted line borders) of the 2.5-30 sample cross-section. These groove defects are arranged along the stir direction and are generated by both the high temperatures and the abnormal stirring reached during the FSP. Fashami et al. [40], who investigate the defects formed during the FSP of an AZ91 alloy, confirmed the same statements. For the same reasons (Table 2), the 3.0-30 samples are characterized by a greater amount of flash formation and bigger groove defects.

The stirred zone (SZ) and the thermomechanically affected zone (TMAZ) are separated from the base material (BM) by the heat-affected zone (HAZ) that experiences only heating and cooling and no deformation [40,41]. The HAZ is close to the white dotted line. The BM (Figure 3b) is characterized by α-Mg grains surrounded by the eutectic phase mainly constituted by α-Mg and β-Mg_17_Al_12_ phases; other phases were detected in the HPDC samples such AlMn-type and MgZn-based phases other than Mg_17_Al_12_ [23]. The β phase is an intermetallic compound having an α-Mn-type cubic unit cell, whereas the matrix phase has the hexagonal closed packed (hcp) structure. In this alloy, precipitation may occur either continuously or discontinuously: discontinuous precipitates form as alternate plates of the secondary phase and near the equilibrium matrix phase at high-angle grain boundaries; continuous precipitates form in all the remaining regions of the supersaturated matrix [41,42]. In the SZ (Figure 3c), the grain boundaries are different from those in the BM: the coarse intermetallic network (by α-Mg and β-Mg_17_Al_12_ phases) surrounded grains, fractured in smaller parts, or partially dissolved due to the temperature increases during the friction stir process [23,43]. As a matter of fact, the rapid dissolution of the β-Mg_17_Al_12_ phase is promoted by the high values of the diffusion kinetics induced by the FSP [32]. Figure 3c also shows material flow patterns within the SZ because of the changes in grain dimensions, as discussed in [27].

**Figure 4:**
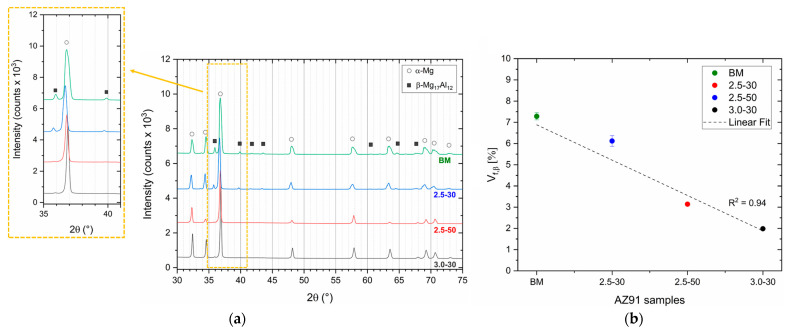
(**a**) XRD spectra performed on BM and FSPed regions of 2.5-30, 2.5-50, and 3.0-30 samples. (**b**) Volume fraction of β-Mg_17_Al_12_ in BM and FSPed regions of 2.5-30, 2.5-50, and 3.0-30 samples.

The XRD spectra (Figure 4a) confirm the β-Mg_17_Al_12_ phase dissolution in the SZ of the FSPed samples because of the reduction in terms of the β-peak intensity. The percentage of the volume fraction $\left(V_{f,\beta }\right)$ indeed decreases from 7.30 ± 0.2% in the BM to a minimum of 2.0 ± 0.1% in the 3.0-30 samples, as shown in Figure 4b. Simultaneously, the increment of the solute concentration in the α-Mg lattice, as well as the severe plastic deformation induced by the tool motion, cause the α-peak shift in the XRD patterns (Figure 4a) [43,44]. The rise in solute atoms in the nugget is also confirmed by the electrical conductivity measurements discussed in our previous works [23,43].

**Figure 5:**
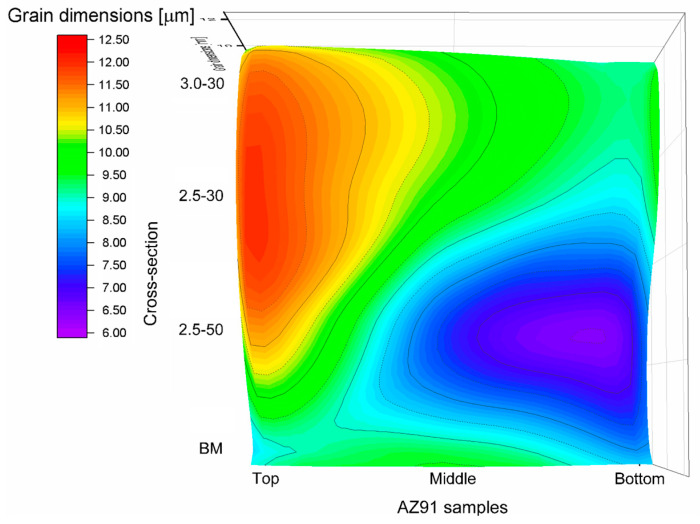
Grain dimensions at top, middle, and bottom regions for unprocessed BM and FSPed (2.5-50, 2.5-30, and 3.0-30) AZ91 samples. The *z*-axis is represented by the colour map titled “Grain dimensions [μm]”.

The α-Mg grain size was measured by the linear intercept method at the top, middle, and bottom of the nugget section, and their average dimension is shown in Figure 5 as a function of the friction stir process parameters. The results show that the mean grain dimensions were quite comparable at the top, middle, and bottom, except for the 2.5-50 sample; in this case, smaller grains were observed because of the lower heat input during the process. An extensive discussion related to both the grain growth and dynamic recrystallization process, which occurred in the SZ, was reported in [45]. Unlike the results published in the literature [46,47], the friction heating developed during the severe plastic deformation does not confer grain refinement in HPDC AZ91 samples (see BM in Figure 5). Nevertheless, a decrement in grain dimensions was observed by increasing the advancing speed from 2500 to 3000 rpm. The same results are obtained by Asadi et al. [48], who analysed an FSPed ultrafine-grained AZ91 alloy.

**Figure 6:**
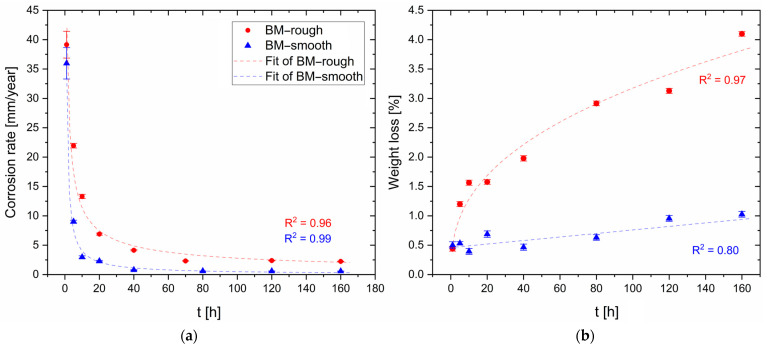
Corrosion rate (**a**) and weight loss (**b**) for BM-rough and BM-smooth HPDC samples.

Figure 6a shows the corrosion rates, calculated with Equation (1), of unprocessed AZ91 platelets with as-cast (BM-rough, Figure 1b) and smoother surfaces (BM-smooth, Figure 1b). In the former case (Figure 2a), the CR exponentially decreases from 39.1 ± 2.3 mm/year at 1 h of immersion time to 6.9 ± 0.2 mm/year at 20 h, and then it tends to settle at around 2.5 ± 0.1 mm/year from 40 h to 160 h. In the latter case, i.e., considering the BM-smooth sample (Figure 2b), CR starts from similarly high values, 36.0 ± 2.7 mm/year at 1 h, but it reaches 3.0 ± 0.2 mm/year just after an immersion time of 10 h. The CR continues to be lower than BM-rough conditions up to 160 h of corrosion time, where it is already stabilized at 0.6 ± 0.1 mm/year. This rapid corrosion attack was also reported by Sahu et al. [3] who analysed the CR of polished AZ91 samples in 3.5% NaCl solution. The same authors reported CR values of the AZ91 alloy in the range of 0.02–0.22 mm/year due to the low surface roughness values conferred by the polishing process and due to the protective behaviour of the hydroxide compounds formed during the corrosion process. On the other hand, despite the polished surface of AZ31 samples, Saikrishna et al. [27] reported a CR value of 4 mm/year after an immersion time of 72 h in 0.9% NaCl solution. Lastly, the high CR values conferred by the short-time tests can present misleading results given that AZ91 alloy forms a passive layer, as confirmed by standard G31 [38]. As a matter of fact, the co-presence of a passive layer and several localized and small anodic regions (pits formed due to the breakdown effects induced by Cl^−^ on the oxide layer) exponentially increases the corrosion attack. The CR obtained after long-time tests can indeed represent more realistic conditions of AZ91 samples because the absence of small pits does not continue to concentrate the corrosion attack. Figure 6b shows the percentage of weight loss during the corrosion period for BM-rough (red symbols) and BM-smooth (blue symbols) samples. The systematic increments of weight loss percentage with the corrosion time of both reflect the different values of the CR (Figure 6a). As a matter of fact, the BM-rough samples exhibit an exponential increment ($CR=0.95t^{0.39}$, $R^{2}=0.97$) while the BM-smooth ones show a linear growing trend (CR=0.005t+0.004, $R^{2}=0.80$) with time (t). The same trends were obtained by Mitchell et al. [49] who tested the AZ31 alloy in a salt fog having 3.5wt.% of NaCl. From the obtained results (Figure 6), it is possible to conclude that the smooth surfaces form an unbroken and more stable passive layer, which increases the corrosion resistance, compared to rougher surfaces. This statement was demonstrated through the potentiodynamic polarization curves performed by [36,50]. Given that BM-rough and BM-smooth samples are characterized by the same microstructural features (Figure 3b), the grain dimensions (Figure 5), the β-Mg_17_Al_12_ morphology and its distribution do not obviously promote variation in the corrosion resistance.

**Figure 7:**
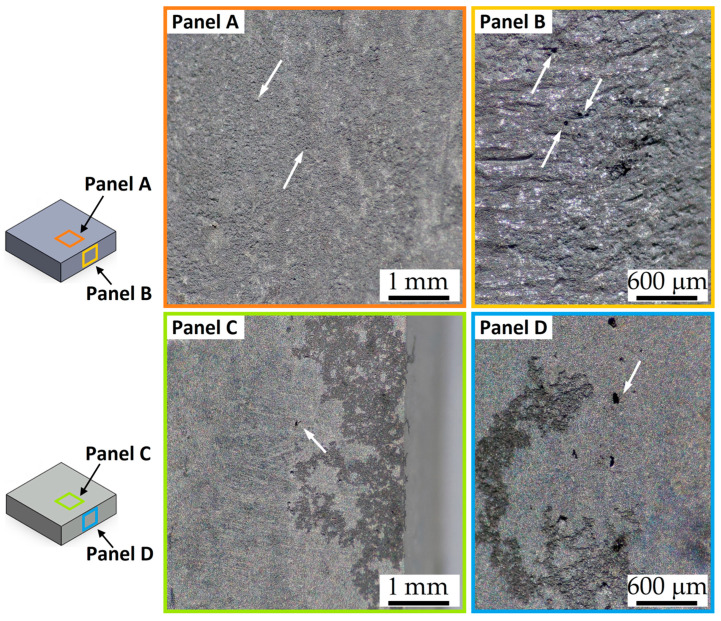
Stereo microscope images of the BM-rough (Panels **A**,**B**) and BM-smooth (Panels **C**,**D**) samples after an immersion time of 10 h in 1M NaCl solution, at magnifications of 8× and 45×. White arrows indicate pits. The CR values are 13.3 ± 0.4 mm/year and 3.0 ± 0.3 mm/year for BM-rough (Panels **A**,**B**) and BM-smooth (Panels **C**,**D**) samples, respectively.

**Figure 8:**
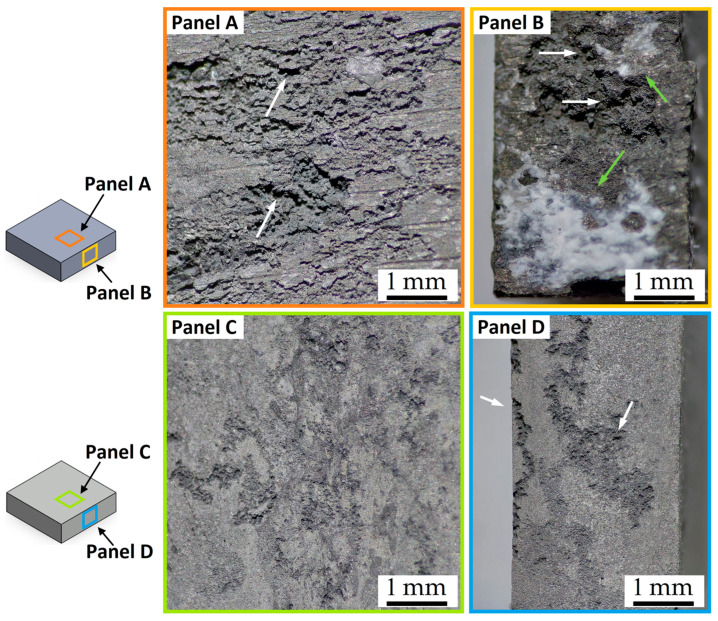
Stereo microscope images of the BM-rough (Panels **A**,**B**) and BM-smooth (Panels **C**,**D**) samples after an immersion time of 120 h in 1M NaCl solution, at magnifications of 8× and 25×. The green and white arrows indicate the corrosion products and pits, respectively. The CR values are 2.4 ± 0.1 mm/year and 0.6 ± 0.1 mm/year for BM-rough (Panels **A**,**B**) and BM-smooth (Panels **C**,**D**) samples, respectively.

The differences in CR and weight loss percentage are observable in Figure 7 where BM-rough (Panels A, B in Figure 7) and BM-smooth (Panels C, D in Figure 7) samples were compared after an immersion time of 10 h. In both cases, the corrosion effects are more pronounced in the smaller areas exposed to the NaCl solution. Kim et al. [51] defined the dimensions of the contact area as a critical factor for determining the corrosion behaviour of Mg alloys. The same authors also revealed a decreasing trend of CR values and, consequently, an increment of weight loss percentage, as also obtained in Figure 6. The higher number of pits, as well as the extended corrosion regions, on BM-rough (Panels A,B in Figure 8) than on BM-smooth (Panels C, D in Figure 7) samples confirms the higher passivation tendency, pitting, and general corrosion resistances of the smoother samples. Walter et al. [36] showed the same results by evaluating the pitting potential versus the surface roughness of sand-cast AZ91 alloy using a 0.5wt.% NaCl solution. In addition, it underlines that pitting corrosion governs the corrosion phenomena of unprocessed AZ91 samples. As a matter of fact, the magnesium dissolution tends to passivate the corded parts due to the increase in local pH, but the presence of Cl^−^ ions breaks down the passive layer and triggers the pitting corrosion [6].

The presence of pits and their depth increase with the immersion time in 1M NaCl solution (Figure 8) despite the reduction in terms of CR (Figure 6a). In addition, the smallest contact areas continue to intensify the corrosion behaviour with respect to the larger ones, as well as the surface roughness by comparing BM-rough (Panels A,B in Figure 8) and BM-smooth (Panels C,D in Figure 8) samples. At the same time, the roughened samples (Panel B in Figure 8) had the highest amount of corrosion products, as indicated by the green arrows and maintained by Mitchell et al. [49]. The same authors revealed the presence of a flower-like oxide in every pit, a film formed by MgO and brucite (Mg(OH)_2_) components thanks to the chemical reactions already presented in the Introduction section. On the other hand, Saikrishna et al. [27] affirmed that the presence of Cl^−^ ions suggests the formation of magnesium chloride on AZ31 samples immersed in 0.9wt.% NaCl solution. Several studies [5,52] suggested that magnesium oxide/hydroxide, formed during the previous redox reaction, is converted to magnesium chloride, as follows:

Mg(OH)_2_ + Cl^−^ → MgCl_2_ + 2OH^−^

where OH^−^ ions continue to accelerate the corrosion process. In addition to the environmental conditions, both the chemical composition and microstructure of the Mg alloy greatly influence its corrosion resistance. Simultaneously, Zhang et al. [53] associated the white corrosion products (Panel B in Figure 8) on AZ91 with brucite and nesquehonite (MgCO_3_·3H_2_O) due to the CO_2_ dissolution in the NaCl solution and the consequent reaction with the Mg hydroxide, as follows:

Mg(OH)_2_ + 2CO_2_ → MgCO_3_ + H_2_O

MgCO_3_ + 3H_2_O → MgCO_3_·3H_2_O

From a microstructural point of view, the corroded surfaces shown in Figure 7 and Figure 8 were promoted by the micro-galvanic couples between the α-Mg matrix and the β-Mg_17_Al_12_ eutectic, as widely discussed both in the literature [2,14,15] and in the Introduction section.

**Figure 9:**
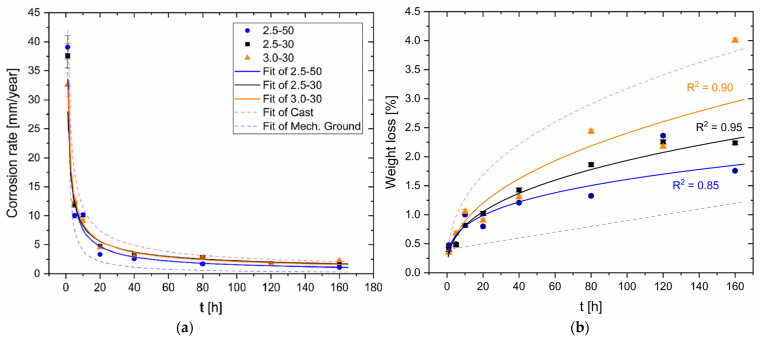
Corrosion rate (**a**) and weight loss (**b**) for FSPed-rough samples.

Figure 9 shows the CR (Figure 9a) and the weight loss percentage (Figure 9b) of FSPed-rough (Figure 1b) samples. The red and blue dotted lines, which indicate the fits of BM-rough and BM-smooth samples (see Figure 6), form a domain where the CR values and the weight loss percentage of FSPed-rough samples are included, respectively. In terms of CR (Figure 9a), the FSPed samples follow the same exponential decreasing trends as the unprocessed ones. The same trends are also discussed by Bobby Kannan et al. [16], who investigated the corrosion phenomena on FSP AZ31 alloy in a simulated body fluid (SBF) solution. The CR decreases from values in the range of 31–44 mm/year at 1 h to values lower than 5 mm/year after an immersion time of 20 h. They consequently settle to rates in the range of 1.2–2.8 mm/year. In this scenario, 2.5-50 samples show CR values lower than those exhibited by 2.5-30 and 3.0-30 samples of about 0.7 ± 0.1 mm/year and, obviously, lower values of mass loss (Figure 9b). Saikrishna et al. [27] reported CR values in the range of 4.63–9.90 mm/year for the AZ31 samples FSPed with a rotational rate of 1100 rpm and an advancing speed of 100 mm/min (*ω*/*v* = 11 r/mm). It is possible to conclude that the FSP increases the corrosion resistance with respect to the BM-rough samples.

**Figure 10:**
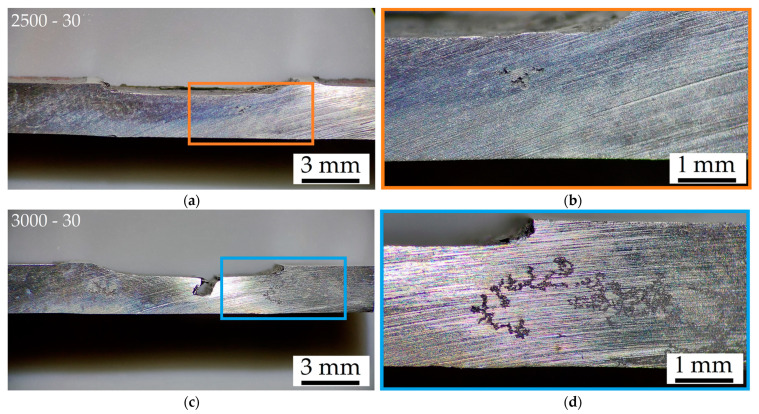
Stereo microscope images of 2.5-30-rough (**a**,**b**) and 3.0-30-rough (**c**,**d**) samples after 1 h of immersion time in 1M NaCl solution, at magnifications of 8× (**a**,**c**) and 25× (**b**,**d**). The CR values are 39.1 ± 1.9 mm/year and 32.6 ± 2.0 mm/year for 2.5-30-rough (**a**,**b**) and 3.0-30-rough (**c**,**d**), respectively.

Stereo microscope images shown in Figure 10 highlight that corrosion effects vary in relation to the friction stir process parameters (Table 2), despite the similar CR values (Figure 10a). As a matter of fact, the corroded zones are always close to the board of the SZ (Figure 3a) both in 2.5-30-rough (Figure 10a,b) and in 3.0-30-rough (Figure 10c,d) samples. In the former case, the pits are formed in the HAZ, while in the latter one, the corrosion processes took place in the BM adjacent to the HAZ due to the different microstructural features in terms of both grain dimensions (Figure 5) and β-Mg_17_Al_12_ distribution (Figure 3, Figure 4). Firstly, the number of grain boundaries, which is directly related to the grain dimensions, influences the corrosion behaviour because they act as cathode and the grain centre as anode [19,20]. Therefore, a variation in grain size modifies the micro-galvanic couple [4,16,21,22,32]. In addition, a high grain boundary density can increase surface activity and, consequently, promote a rapid formation of a passivating layer. Bobby Kannan et al. [14] revealed an increment of corrosion resistance in FSPed AZ31 samples which were immersed in SBF thanks to grain refinement, unlike Saikrishna et al. [27] who affirmed that the refinement (from 16.4 ± 6.8 μm to 3.2 ± 1.2 μm) may improve the corrosion resistance of FSPed AZ31 samples in 0.9% NaCl solution. The more serious localized corrosion conferred by sodium chloride solution rather than SBF can explain this different corrosion behaviour [54]. Therefore, the 2.5-30 sample shows the corroded area within the FSPed region (Figure 10b) due to both the higher grain dimension (Figure 5) and the more fractured eutectic phase with respect to the BM. In this zone, the more uniform distribution of β-Mg_17_Al_12_ confers the barrier effects on corrosion phenomena as discussed in the Introduction section.

**Figure 11:**
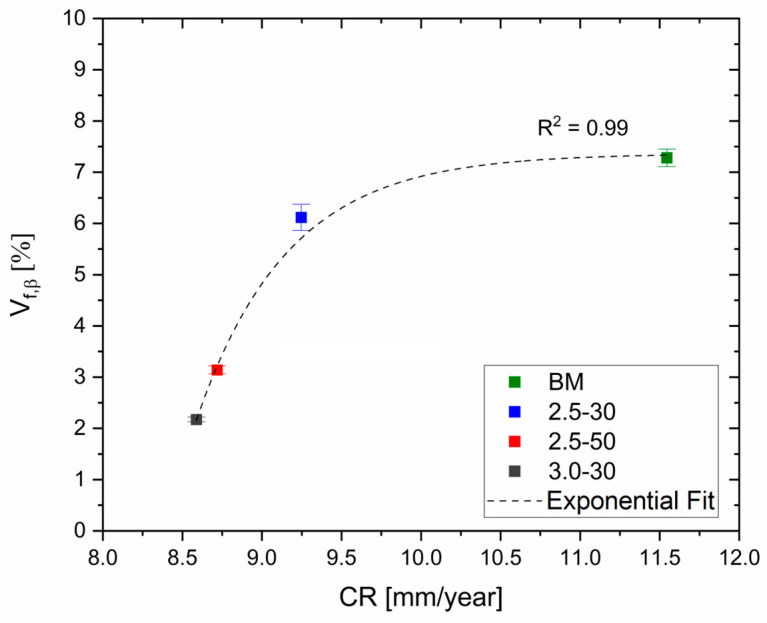
Volume fraction of β-Mg_17_Al_12_ eutectic versus mean values of CR.

In more detail, pits are formed in the HAZ because it may also present a smaller amount of wt.% Al in solid solution with respect to the SZ. Secondly, the fragmented β-Mg_17_Al_12_ eutectic, still present (Figure 4) in the 2.5-30-rough sample, intensifies the micro-galvanic couple with the α-Mg matrix with respect to the 3.0-30-rough one [15]. On the other hand, the near absence of the eutectic phase in 3.0-30-rough samples increases the wt.% Al in the α-Mg matrix and, consequently, the corrosion resistance of its SZ zone (Figure 10d) [11,55]. This statement was supported by the correlation between the mean values of the CR and the β-Mg_17_Al_12_ volume fraction shown in Figure 11. The CR decreases by increasing the amount of dissolved eutectic in the matrix.

**Figure 12:**
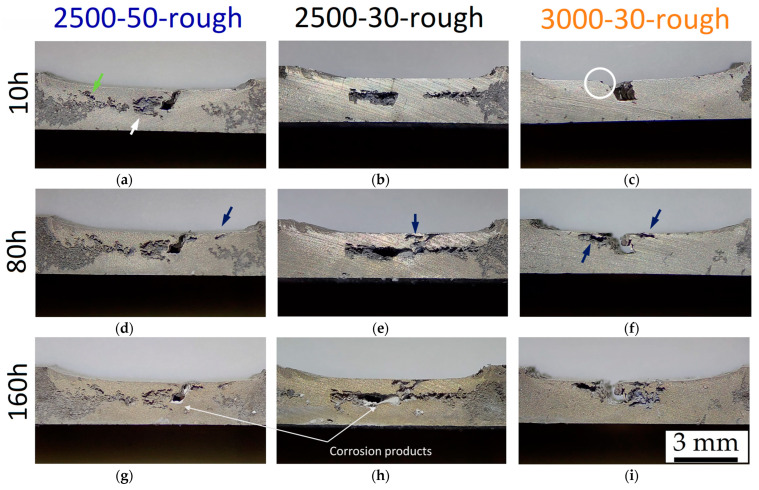
Corrosion effects after 10 h (**a**–**c**), 80 h (**d**–**f**), and 160 h (**g**–**i**) of immersion time in NaCl solution on FSPed cross-section of 2.5-50-rough (**a**,**d,g**), 2.5-30-rough (**b**,**e**,**h**), and 3.0-30-rough (**c**,**f**,**i**) samples. Stereo microscope images are at magnifications of 8×.

The 2.5-50-rough sample does not show evident corroded zones after 1 h of immersion time through stereo microscope observations. Therefore, the weighted mass loss (~2.6 mg), which confers a CR of 39.1 ± 1.9 mm/year, may be concentrated within the groove defects or in small pits where the corrosion attack is localized. The corrosion effects are indeed observable after 10 h of immersion time both in the BM and FSPed region (Figure 12a), in which deeper pits are more localized and already formed after 5 h of immersion time. In addition, a greater corrosion attack is localized on the centre of the nugget (white arrows) and on the TMAZ/HAZ (green arrows), regions where the grain dimensions and β–eutectic phase govern the corrosion phenomena. These corrosion effects are more impactful in 2.5-30-rough samples (Figure 12b). After an immersion time of 10 h, the 3.0-30-rough sample (Figure 12c) always had more intensified corrosion phenomena in the BM and the corrosion activation on the edge of the groove defect (white circle). Increasing the immersion time causes both an increment of the corrosion effects in the sites previously corroded and the activation of new ones (blue arrows in Figure 12d–f).

**Figure 13:**
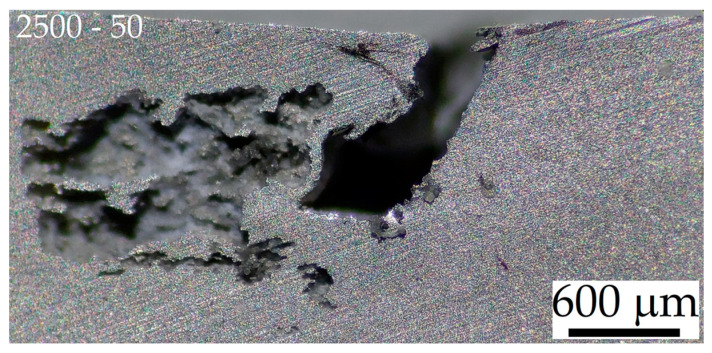
Stereo microscope image of the FSPed surface of 2.5-50-rough sample (Figure 12d), at magnification of 45×, after an immersion time of 80 h in 1M NaCl solution.

The centre of the nugget zones of 2.5-50-rough and 2.5-30-rough samples show a higher amount of corrosion effects probably due to the impacts induced on the microstructure by the material flow during the FSP. In other words, the typical onion ring structure, which characterizes the transverse section of the SZ, and the differences in the grain dimension between the bands of the same onion rings (see the results presented in our previous work [23]) promote a corrosion attack more localized in the nugget regions, as also supported by Zeng et al. [56]. As a matter of fact, the same authors affirmed that a macro-galvanic corrosion is triggered in the region where two adjacent layers are characterized by different grain dimensions. Figure 13 shows a magnified area of Figure 12d in which the groove defect seems not to trigger the corrosion process given that it is separated from the corroded area. The same results can be observed in Figure 12c,f and will be exhibited by FSPed-smooth samples.

**Figure 14:**
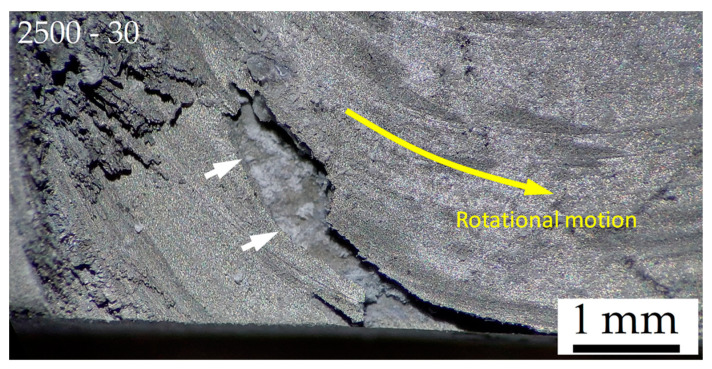
Stereo microscope image of the FSPed surface of 2.5-30-rough sample (Figure 12e), at magnification of 45×, after an immersion time of 80 h in 1M NaCl solution.

The corrosion phenomena in 2.5-30-rough samples continue to follow the HAZ as well (see Figure 12a,b). Simultaneously, the possible presence of defects on the FSPed surface (Figure 2a), as well as the corrosion process in the nugget zone, promotes the corrosion attacks and the consequent material loss on the same FSPed surface (white arrows in Figure 12 and Figure 14). From a morphological point of view, the corrosion phenomena seem to follow the material flow direction induced by the FSP (yellow arrow in Figure 14).

The 3.0-30-rough sample (Figure 12f) continues to show corrosion phenomena in BM, but the presence of the groove defect triggers; in this case, the corrosion process in the SZ. At the same time, the corroded site indicated by the upper blue arrow in Figure 12f seems to induce the corrosion process in the nugget zone. In all cases, the increment of the immersion time in 1M NaCl solution keeps on corroding the weakened sites (Figure 12g–i).

**Figure 15:**
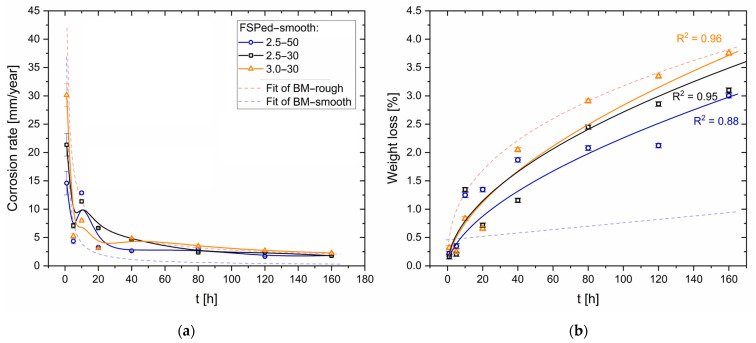
Corrosion rate (**a**) and weight loss (**b**) for FSPed-smooth samples.

**Figure 16:**
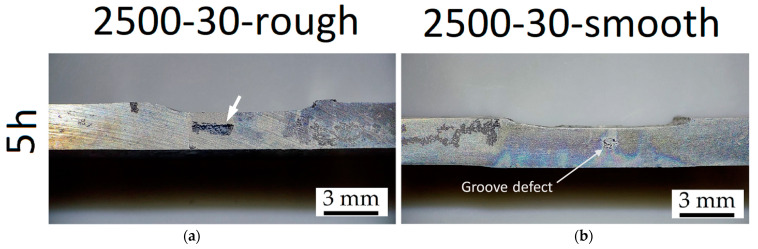
Corrosion effects after 5 h of immersion time in NaCl solution on FSPed cross-section of 2.5-50-rough (**a**) and 2.5-30-smooth (**b**). Stereo microscope images are at magnifications of 8×. The CR values are 11.9 ± 0.5 mm/year and 4.37 ± 0.5 mm/year for 2.5-30-rough (**a**) and 2.5-30-smooth (**b**), respectively.

**Figure 17:**
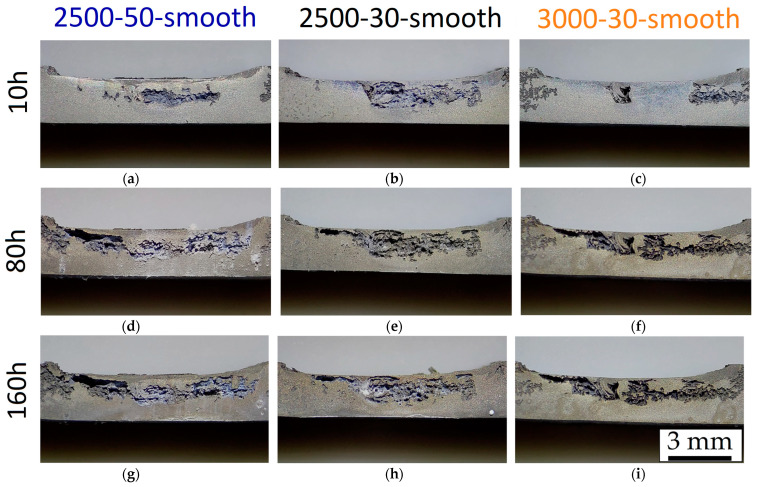
Corrosion effects after 10 h (**a**–**c**), 80 h (**d**–**f**), and 160 h (**g**–**i**) of immersion time in NaCl solution on FSPed cross-section of 2.5-50-smooth (**a**,**d,g**), 2.5-30-smooth (**b**,**e**,**h**), and 3.0-30-smooth (**c**,**f**,**i**) samples. Stereo microscope images are at magnifications of 8×.

Figure 15 shows the CR (Figure 15a) and the weight loss percentage (Figure 15b) of the FSPed-smooth samples. As already highlighted by BM-rough (red dotted line, Figure 15a) and BM-smooth (blue dotted line, Figure 15b) samples, the CR values decrease with the surface roughness. The statement still takes effect on FSPed-rough (Figure 9a) and FSPed-smooth (Figure 15a) samples until 5 h of immersion time given that CR values of FSPed-smooth samples are lower (−47%) than those shown by the FSPed-rough ones. The CR successively reached values of the relative maximum (7.5–12.5 mm/year) at 10 h of immersion time for each FSPed-smooth condition, and then it settled down around 1.6–2.6 mm/year, following the BM-rough trend (red dotted line, Figure 15a). The percentages of weight loss retrace the exponential increments already discussed for FSPed-rough in Figure 9b. On the other hand, if the values of weight loss in the first two steps of the corrosion test are even lower than the BM-smooth case, the percentages overstep the FSPed-rough (Figure 9b) after 10 h of immersion time. Therefore, it is possible to observe that FSP slightly increases the corrosion resistance compared to the BM-smooth condition given that they are characterized by the same values of S_a_ (see discussion of Figure 2). This may be true until 10 h of immersion time, where the reached CR values are also higher than those shown by FSPed-rough samples. As a matter of fact, the smoother surface of FSPed-smooth samples delayed the strongly localized corrosion attack in the nugget zones from 5 to 10 h, as confirmed by Figure 16 and Figure 17a,b.

Figure 16a shows the localized corrosion in the nugget zone (white arrow) of the 2.5-30-rough sample already after 5 h of immersion time (compared to Figure 12b), while Figure 16b exhibits the localized corrosion attack only in the BM of the 2.5-30-smooth sample.

The corrosion attack becomes more localized in the nugget zone of 2.5-30- and 2.5-50-smooth samples (Figure 17a,b) after 10 h of immersion time. Figure 17c indeed shows the 3.0-30-smooth samples where their corrosion behaviour retraces that described in Figure 12c in which the FSP seems to confer an increase in the corrosion resistance of the SZ and, consequently, a more localized corrosion attack in the TMAZ/HAZ zones.

By increasing the immersion time, the CR reached values comparable to those shown by FSPed-rough samples (compare Figure 9a to Figure 15a); the greater stability of the passive layer on the smooth surface than on the rough one may confirm, firstly, the higher corrosion resistance in FSPed-smooth samples (<10 h) and, secondly, increase the corrosion attack in the SZ after 10 h of immersion time due to its cathodic behaviour [27,36,57]. As a matter of fact, FSPed-smooth samples show BM regions less corroded by the 1M NaCl solution compared to the same zones exhibited in Figure 12d–i.

From the present study, the nonuniform microstructure of FSPed samples, as well as their surface roughness, affects the corrosion resistance of HPDC AZ91 samples. As a matter of fact, the BM generally shows corrosion phenomena less localized than the FSPed samples, especially for 2.5-30 and 2.5-50. The higher amount of β-phase dissolution in 3.0-30 and the consequent increase of wt.% Al in α-Mg matrix raise the corrosion resistance of the FSPed zone regardless of the different surface roughness. On the other hand, the greatest stability of the passive layer in the BM regions of FSPed-smooth samples, and its cathodic behaviour, seems to localize and intensify the corrosion attacks in the FSP regions (t > 10 h). As also suggested by Saikrishna et al. [27], if the targeted application of the FSPed HPDC AZ91 alloy is within a corroding environment in which the Cl^−^ ions are absent or in a negligible amount, the nonuniform microstructure can be suggested, also considering its mechanical properties [43,44,58]. For biodegradable applications in the biomedical field or in other application fields containing a higher amount of chloride ions, the uniform microstructure, as well as the coating deposition, could be preferred.

## 4. Conclusions

Corrosion responses of HPDC AZ91 alloys in the as-cast and friction stir processed conditions were investigated after immersion tests in 1M NaCl solution. The following conclusions can be drawn:

1. The corrosion rate of the HPDC AZ91 alloy decreases in samples with lower surface roughness. The smooth samples (S_a_ = ~0.07 μm) are indeed characterized by slower corrosion rates (<1 mm/year) than rougher samples (S_a_ = ~0.50 μm), which indeed show a higher number of pits.
2. FSP improves the corrosion resistance of HPDC samples due to the fracture and dissolution of β-Mg_17_Al_12_ eutectic and the resulting increase in wt.% Al in the α-Mg matrix. These phenomena play a predominant role in corrosion behaviour with respect to the grain size. On the other hand, FSPed-smooth samples show better corrosion resistance than the FSPed-rough thanks to the greatest stability of the passive layer (t < 10 h). After 10 h of immersion time, the corrosion attack becomes strongly localized in the SZ regions (t > 10 h) because a macro-galvanic couple between the passive layer and the SZ may be formed.
3. The HPDC samples friction stir processed at 2500 rpm and 50 mm/min show better corrosion resistance than the other conditions.
4. The corrosion phenomena are not influenced by the groove defects, but the typical onion ring structure seems to promote a localized corrosion attack in the nugget zone.

## Figures and Tables

**Table 1:** Chemical composition (wt.%) of AZ91 plates.

Mg	Al	Zn	Mn	Si	Cu	Ni	Fe	Be
Bal.	8.90	0.79	0.28	0.0093	0.0013	0.001	0.0017	3.6 ppm

**Table 2:** Designation and temperatures measured behind the pin during the FSP.

Designation	[rpm]-[mm/min]	*w/v* [r/mm]	Temperature [°C]
2.5-50	2500-50	50	198
2.5-30	2500-30	83.3	220
3.0-30	3000-30	100	265

## Data Availability

Not applicable.

## Abbreviations

BM	Base Material
CR	Corrosion Rate
HPDC	High-Pressure Die Cast
FSP	Friction Stir Process
SZ	Stirred Zone
TMAZ	Thermomechanically Affected Zone
XRD	X-ray Diffraction
